# ^18^F-FDG PET/CT for Identifying the Potential Primary Diseases and Predicting Prognosis of Secondary Hemophagocytic Lymphohistiocytosis in Children

**DOI:** 10.1155/2022/4849081

**Published:** 2022-04-16

**Authors:** Ang Wei, Xia Lu, Honghao Ma, Hongyun Lian, Xu Yang, Liping Zhang, Dong Wang, Sitong Chen, Qing Zhang, Zhigang Li, Rui Zhang, Jigang Yang, Tianyou Wang

**Affiliations:** ^1^Hematology Center, Beijing Key Laboratory of Pediatric Hematology Oncology, National Key Discipline of Pediatrics (Capital Medical University), Key Laboratory of Major Disease in Children, Ministry of Education, Beijing Children's Hospital, Capital Medical University, National Center for Children's Health, Beijing 100045, China; ^2^Nuclear Medicine Department, Beijing Friendship Hospital Affiliated to Capital Medical University, Beijing 100050, China; ^3^Hematologic Disease Laboratory, Beijing Pediatric Research Institute, Hematology Center, Beijing Key Laboratory of Pediatric Hematology Oncology, National Key Discipline of Pediatrics (Capital Medical University), Key Laboratory of Major Disease in Children, Ministry of Education, Beijing Children's Hospital, Capital Medical University, National Center for Children's Health, Beijing 100045, China

## Abstract

Hemophagocytic lymphohistiocytosis (HLH) is a rare, potentially fatal illness, which can be divided into primary HLH (pHLH) and secondary HLH (sHLH). pHLH can be driven by genetic defections. Moreover, the sHLH is usually be triggered by malignancy or non-malignancy diseases. Sixty-two newly diagnosed sHLH patients with known etiology and those who underwent ^18^F-FDG PET/CT examination from July 2018 to December 2020 were retrospectively analyzed. They were divided into malignancy-associated HLH (M-HLH, *n* = 13) and non-malignancy-associated HLH (NM-HLH, *n* = 49). The metabolic parameters of the liver (Li), spleen (Sp), bone marrow (BM), lymph nodes (LN), and their ratios to the liver background (LiBG) and mediastinum (M) were compared between two groups. These metabolic parameters were evaluated for correlation with laboratory parameters and prognostic parameters. We found that the SUV_max_-LN/Sp/Li and SUV_mean_-Sp in M-HLH were significantly higher than those in NM-HLH (*P*=0.031, 0.035, 0.016,  and 0.032). The malignant disease should be considered when SUVmax-LN was higher than 4.41 (sensitivity 61.5%, specificity 81.6%). Hypermetabolic lesions in extranodal organs were more likely to occur in M-HLH than in NM-HLH (*P*=0.011). IFN- *γ* was positively correlated with SUVmax-BM/Li/Sp and SUVmean-BM/Li/Sp (*P* < 0.05). Ferritin, sCD25, IL-6, and IL-10 were positively correlated with SUVmax-Sp and SUVmean-Sp (*P* < 0.05). In Epstein–Barr virus-associated HLH (EBV-HLH), the SUV parameters of bone marrow were significantly correlated with a poor 2-week treatment response, overall survival, and event-free survival (*P* < 0.05). We conclude that some ^18^F-FDG PET/CT metabolic parameters can help identify the etiology of sHLH in children and provide directions for further inspection. The malignant disease should be considered when the SUVmax-LN is higher than 4.41 and hypermetabolic lesions occur in extranodal organs. In EBV-HLH, a higher SUV of bone marrow is associated with a poorer prognosis.

## 1. Introduction

Hemophagocytic lymphohistiocytosis (HLH), also called hemophagocytic syndrome, is a rare, potentially fatal illness characterized by an impaired natural killer (NK) cell and cytotoxic T-cell function [1]. HLH often occurs in adolescents and children. It can be categorized into two distinct forms: primary or familial HLH (FHL) and secondary HLH (sHLH). sHLH can be triggered by different stimuli, including infection, malignant tumors, autoimmune disorders, and so on [2]. Infection is the most common trigger of sHLH in children, especially the Epstein–Barr Virus (EBV) [3]. Identifying the primary underlying disease is even more crucial in the case of sHLH to start proper treatment. Up to now, only a few reports have described the value of fluorine-18 fluorodeoxyglucose (^18^F-FDG) positron-emission tomography/computed tomography (PET/CT) in etiology identification and prognosis prediction in the adult with sHLH [4]. However, few studies reported the role of ^18^F-FDG PET/CT in children. Therefore, the study aims to assess the value of ^18^F-FDG PET/CT in the etiology identification and prognosis prediction in children with sHLH.

## 2. Materials and Methods

### 2.1. Patients

Retrospectively, single-center data were collected from 66 patients who were newly diagnosed with sHLH and underwent ^18^F-FDG PET/CT examination from July 2018 to December 2020 in Beijing Children's Hospital, Capital Medical University. This study was conducted in accordance with the Declaration of Helsinki and approved by the Institutional Review Board (IRB) of Beijing Children's Hospital, Capital Medical University. All patients were asked to sign informed consent.

### 2.2. Diagnostic and Evaluation Criteria

The sHLH was defined as a patient meeting HLH-94 diagnostic criteria without mutations of known pHLH-associated genes [5]. The efficacy for the treatment of HLH was assessed according to the criteria proposed by the Midwest Collaboration Group of the United States [6]. The evaluation depends on the following 6 quantifiable symptoms and laboratory biomarkers of HLH: (1) Serum soluble cluster of differentiation 25 (sCD25); (2) ferritin; (3) complete blood count (CBC); (4) triglyceride; (5) presence of hemophagocytosis in pathology specimen; and (6) level of consciousness (documented in clinical exams in patients with the central nervous system (CNS) involvement. A complete remission (CR) is defined as normalization of all quantifiable symptoms and laboratory biomarkers of HLH, while a partial remission (PR) is defined when at least 25% improvement in two or more quantified symptoms and laboratory biomarkers, and a PR also requires every single item to meet the specific criteria as below: (1) sCD25 is greater than 1.5 fold decrease; (2) ferritin and triglyceride decrease at least 25%; (3) without transfusion: For patients with an initial absolute neutrophil count(ANC) of less than 500 cells/ml, ANC increases by at least 100% to greater than 500 cells/ml; for patients with an initial ANC of 500–2000 cells/ml, ANC increases by 100% to more than 5000 cells/ml; and (4) for patients with transaminitis with an ALT greater than 400U/L, ALT decreases at least 50%. A no remission (NR) is defined when PR criteria are not met. Briefly, the treatment response was categorized as no remission (NR), partial remission (PR), and complete remission (CR).

### 2.3. Clinical and Laboratory Data

Demographic characteristics were collected, including age and gender. Laboratory findings included ANC, platelet, hemoglobin, fibrinogen, serum ferritin, triglyceride, sCD25, NK cell activity, interferon (IFN)-*γ*, tumor necrosis factor (TNF-*α*), interleukin (IL)-6, and IL-10, which were obtained within one week before the ^18^F-FDG PET/CT study. Treatment outcomes and mortality were collected. The last follow-up date was July 1, 2021. Overall survival (OS) was estimated from diagnosis until death due to any reason or previous contact with the patients. Event-free survival (EFS) was calculated from the date of diagnosis until the date of events, including refractory, relapse, and death.

### 2.4. ^18^F-FDG PET/CT Acquisition

^18^F-FDG PET/CT was conducted using a Siemens Biography 64 HR PET/CT scanner (Siemens Medical Solution, Erlangen, Germany) in Beijing Friendship Hospital, Capital Medical University. Patients should fast for more than 6 hours and have a blood glucose level <11.1 mmol/L. Then, ^18^F-FDG was injected intravenously at a dose of 3.7 MBq/kg. Sixty minutes after injection, a scan from the top of the head to the midthigh was required. The CT scan was obtained at 120 kV, 200 mA, and 3 mm thickness. Then, PET was obtained in a 3-dimensional mode at 2 min/bed. After the attenuation correction with CT data, the iterative method was used to reconstruct the images.

### 2.5. Image Analysis

Two experienced nuclear medicine physicians analyzed 18F-FDG PET/CT images on a dedicated workstation (syngo Multimodality Workstation, Siemens Healthineers). The spherical regions of interest (ROI) were drawn to measure the maximum standard uptake value (SUVmax) and mean standard uptake value (SUVmean) of the liver (Li), spleen (Sp), bone marrow (BM), lymph nodes (LN), liver background (LiBG), and the mediastinum (M). If the FDG uptake was homogeneous in the liver, spleen, or bone marrow, the ROI of Li, Sp, and BM were drawn over the right lobe of the liver, in the middle of the spleen, and in the vertebral body with the highest FDG uptake from thoracic (T10-12) to lumbar(L1-4) vertebrae, with a diameter of 3 cm, 2 cm, and 1 cm, respectively. Otherwise, the ROI was drawn over the hypermetabolic lesion. The ROI of LN was drawn over the lymph node with the highest FDG uptake. The ROI of LiBG was drawn as a 3 cm sphere in the right lobe of the liver generally, avoiding the hypermetabolic lesions. The ROI of M was drawn as a 1 cm sphere in the aortic arch (0.5 cm if age＜10 years). The FDG uptake of the liver, spleen, bone marrow, and lymph nodes was considered elevated when the SUV > 2.5 or SUV > SUV-LiBG. Hypermetabolic lesions were diagnosed with a clear focus with elevated FDG uptake over the peripheral background tissue.

### 2.6. Statistical Analysis

Statistical analyses were performed using IBM SPSS Statistics 24 software (IBM, USA). The continuous variables with a normal or skewed distribution were presented as mean ± standard deviation (SD) or median (interquartile ranges). Categorical variables were presented as numbers (percentages (%)). The *t*-test or the Mann–Whitney test was used to determine differences between numerical variables with a normal or a skewed distribution between two groups, respectively. A comparison between categorical variables was performed using the Pearson *χ*^2^ or Fisher exact test. The ROC curve was used to calculate the cut-off value. *P* < 0.05 indicated a significant difference. ^*∗*^*P* < 0.05, ^*∗∗*^*P* < 0.01.

## 3. Results

### 3.1. General Patient Information

Sixty-six children with sHLH were enrolled in this study, 33 (50%) males and 33 (50%) females, with a median age of 7 years (range, 9 months to 14 years). The underlying causes of sHLH were determined in 62 of the 66 patients whose final diagnosis was established through clinical features and/or biopsy. Among the 62 patients, 13 (21.0%) had malignancies, including 10 lymphomas (Six Hodgkin's lymphomas, 2 Burkitt lymphomas, 1 anaplastic large cell lymphoma, and 1 subcutaneous panniculitis T-cell lymphoma), 1 mixed lineage leukemia, 1 malignant histiocytosis, and 1 angiosarcoma. Forty-nine had nonmalignant diseases, including 20 chronic active Epstein–Barr virus (CAEBV) infections, 21 EBV-HLHs, 4 autoinflammatory diseases, 2 subacute necrotizing lymphadenitises (SNLs), 1 Langerhans cell histiocytosis, and 1 type IV glycogen storage disease. All these patients presented HLH at the disease onset. More information about clinical manifestations and laboratory examinations is shown in Table 1.

### 3.2. ^18^F-FDG PET/CT Characteristics

The ^18^F-FDG PET/CT examination was performed in all children within 1 week after admission. In those sHLH patients, ^18^F-FDG PET/CT demonstrated diffuse, heterogeneous, or focal increased FDG uptake in organs, mostly in lymph nodes (52 cases), spleen (43 cases), and bone marrow (54 cases). Other organs included intracranial (2 cases), lung (15 cases), intestinal (2 cases), kidneys (6 cases), liver (10 cases), adrenal (2 cases), skin or nasal mucosa (7 cases), and muscle (2 cases). The PET metabolic parameters are shown in Table 2.

### 3.3. ^18^F-FDG PET/CT for Detection of Malignancy in sHLH Children

**Figure 1:**
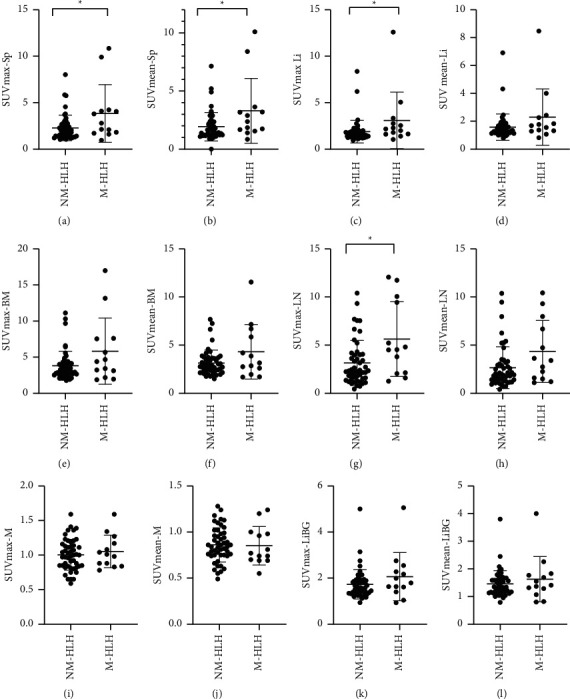
Difference of PET metabolic parameters between M-HLH and NM-HLH. The SUV_max_-LN/Sp/Li and SUV_mean_-Sp in M-HLH were significantly higher than those in NM-HLH (*P*=0.031, 0.035, 0.016,  and 0.032) (a)–(c), and (g). There was no significant difference between the two groups between SUVmax-BM (e) and SUVmean-LN/Li/BM (d), (f), and (h). M-HLH: malignancy-associated HLH; NM-HLH: non-malignancy-associated HLH; SUVmax: maximum standard uptake value; SUVmean: mean standard uptake value; Sp: spleen; Li: liver; BM: bone marrow; LN: lymph nodes; M: mediastinum; and LiBG: liver background.

Four patients with unknown diagnoses were excluded. According to the primary diseases, the other patients were divided into malignancy-associated HLH (M-HLH, *n* = 13) and non-malignancy-associated HLH (NM-HLH, *n* = 49). The SUV_max_-LN/Sp/Li and SUV_mean_-Sp in M-HLH were significantly higher than those in NM-HLH (*P*=0.031, 0.035, 0.016, and 0.032). There was no significant difference in SUV_max_-BM and SUV_mean_-LN/Li/BM between the two groups (Figure 1). We also analyzed the SUVmax and SUVmean ratios of Sp, BM, and LN to M and LiBG between the two groups and found no significant difference (*P* > 0.05).

ROC curves demonstrated that SUVmax-LN > 4.41 (sensitivity 61.5%, specificity 81.6%), SUVmax-Sp > 2.785 (sensitivity 53.8%, specificity 81.6%), SUVmax-Li > 1.965 (sensitivity 61.5%, specificity 79.6%), and SUVmean-Sp > 1.39 (sensitivity 53.8%, specificity 79.6%) were the optimal cut offs for predicting the malignant disease. The sensitivity and specificity of SUVmax-LN > 4.41 were higher than others, and the area under the curve was larger (1.431).

**Figure 2:**
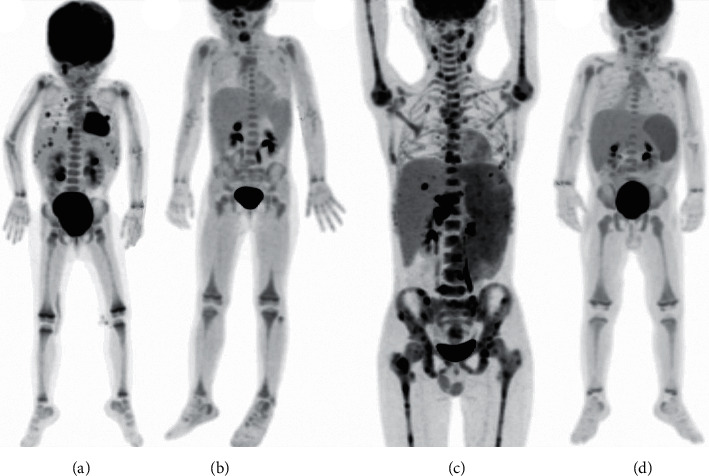
^18^F-FDG PET/CT maximum intensity of sHLH in 4 children. Non-Hodgkin's lymphoma (a), CAEBV (b), EBV-HLH (c, d), presenting as hepatosplenomegaly and increased FDG uptake of the spleen (a–d) and liver (a, c). (a) A 2-year-old girl with multiple enlarged lymph nodes (SUVmax:4.81) and multiple hypermetabolic lesions in extranodal organs, including brain, lungs, liver, and kidney (SUVmax:11.50). The child died three months later after admission. (b) A 3-year-old girl with multiple small lymph nodes (SUVmax:1.68, which was lower than the liver) slightly increased FDG uptake in the bone marrow (SUVmax:1.77). The children survived until the end of follow-up after hematopoietic stem cell transplantation. (c) A 14-year-old boy with focus and defusing increased FDG uptake in the bone marrow (SUVmax:11.13). He died of multiple organ failure. (d) A 2-year-old boy with slightly increased FDG uptake (SUVmax:2.1) in the bone marrow was slightly lower than the liver. He was still alive until the end of the follow-up.

Whether there is a hypermetabolic lesion or not is also one of the critical indicators of PET-CT in differentiating malignant diseases from non-malignant diseases. Our study demonstrated that in the M-HLH group, patients were more likely to have focal hypermetabolism in extranodal organs (*P*=0.011), especially the increased non-physiological focal metabolism of the intestinal tract, skin, and/or nasal mucosa (*P*=0.041 and 0.045) (Table 3). Typically, ^18^F-FDG PET/CT imaging in malignant and non-malignant diseases was shown in Figures 2(a) and 2(b) (Figure 2). In these figures, we can also find that although a higher SUVmax was more suggestive of a malignant process, there was still a significant overlap of the SUVmax (or mean) between malignant and non-malignant conditions.

### 3.4. Correlation between PET Metabolic Parameters and Laboratory Parameters

The results of correlation analysis showed that the IFN-*γ* level had a positive correlation with SUVmax-BM and SUVmean-BM (*r* = 0.259, *P*=0.002 and *r* = 0.238, *P*=0.005). The IFN-*γ* level had a positive correlation with SUVmax-Li and SUVmean-Li (*r* = 0.205, *P*=0.016 and *r* = 0.232, *P*=0.007). The level of serum ferritin, sCD25, IL-6, Il-10, and IFN-*γ* had a positive correlation with SUVmax-Sp (*r* = 0.203, *P*=0.016; *r* = 0.196, *P*=0.022; *r* = 0.234, *P*=0.0193; *r* = 0.193, *P*=0.024; and *r* = 0.179, *P*=0.036). The level of serum ferritin, sCD25, IL-6, Il-10, and IFN-*γ* had a positive correlation with SUVmean-Sp (*r* = 0.213, *P*=0.012; *r* = 0.179, *P*=0.036; *r* = 0.208, *P*=0.014; *r* = 0.210, *P*=0.014; and *r* = 0.217, *P*=0.011). There was no correlation between other PET metabolic parameters and laboratory results.

CNS involvement may occur in patients with sHLH. Among the 66 cases, 14 cases were diagnosed as CNS involvement according to the neurological symptoms, cerebrospinal fluid tests, or neuroradiological examinations. Two patients with an abnormal increase of central FDG uptake indicated by PET-CT were clinically confirmed. Of these 2 cases, 1 case had CNS metastasis of lymphoma and 1 patient had CNS involvement of EBV-HLH.

### 3.5. Prognostic Value of ^18^F- FDG PET/CT in sHLH Children

After a median follow-up of 20.3 months (range 0.13–36.43 months), nine patients had died, six patients gave up treatment at the terminal stage, and five patients relapsed. We analyzed the relationship between the metabolic parameters of ^18^F-FDG PET/CT and the prognosis of HLH and evaluated the treatment response at the 2^nd^, 4^th^, 6^th^, and 8^th^ week, respectively. We found no correlation between the PET metabolic parameters and treatment response, OS, and EFS.

Considering that the abovementioned results may be due to the complex etiology of sHLH, there are significant differences in the prognosis of different diseases. To reduce the difference in prognosis caused by the primary diseases, we reanalyzed the relationship between the prognosis of EBV-HLH, the most common subclass of sHLH, and PET metabolic parameters. All EBV-HLH patients accepted the modified HLH-94 protocol (including methylprednisolone and etoposide). This study found that high levels of SUVmax-Li, SUVmean-Li, SUVmax-BM, and SUVmean-BM were correlated with a poor treatment response (not reaching PR) at 2 weeks (*P*=0.031, 0.008, <0.0001,  and  < 0.0001). In addition, we also found that SUVmax-BM/M ratio, SUVmean-BM/M ratio, SUVmax-BM/LiBG ratio, and SUVmean-BM/LiBG ratio were correlated with a poor treatment response (not reaching PR) at 2 weeks (*P* < 0.0001, <0.0001, 0.003 and 0.019). The high SUVmax-Li, SUVmean-Li, SUVmax-BM, and SUVmax-BM/M ratio correlated with poor OS (*P*=0.011, 0.021, 0.028 and 0.047). The high levels of SUVmax-BM, SUVmax-BM/M ratio, SUVmax-BM/LiBG ratio, and SUVmean-BM/LiBG ratio were correlated with the poor EFS rate (*P*=0.039, 0.028, 0.009,  and 0.020). The ^18^F-FDG PET/CT imaging of two children with EBV-HLH with different prognoses is shown in Figures 2(c) and 2(d) (Figure 2).

## 4. Discussion

HLH is a group of clinical syndromes that leads to abnormal activation and proliferation of lymphocytes and macrophages, with phagocytosis of blood cells and secretion of many cytokines resulting in excessive inflammatory reaction. The main feature is the continuous activation of immune cells, resulting in progressive immune damage. At present, it is believed that sHLH is mainly related to cytokine storms (such as IL-6, IL-10, IFN- *γ*, TNF- *α*, and so on) secondary to various causes. It had rapid progress and a poor prognosis. Early diagnosis and timely treatment are the most effective measures to improve the prognosis of the disease. Therefore, clinicians need to use auxiliary examination to identify the underlying disease and determine the appropriate treatment as soon as possible [7, 8]. ^18^F-FDG PET/CT is a medical imaging technique displaying anatomical morphology and functional metabolism, with high sensitivity and noninvasiveness. It is one of the best methods to diagnose and guide the treatment of hematological tumor diseases [9]. Because fever and enlarged lymph nodes are the first manifestations of HLH in the early stage, and lymphoma is the most common malignant disease in children with sHLH, early ^18^F-FDG PET/CT examination has a particular significance for the detection of the underlying disease in sHLH [10].

Previous studies have found that the whole-body scan of ^18^F-FDG PET/CT can accurately evaluate the range of lesions involved and determine the best location of biopsy, which helps identify the potential causes of sHLH. Sometimes, pathological evidence cannot be obtained due to various reasons. PET-CT can better distinguish the malignant disease from non-malignant diseases, thus providing important guidance for the choice of treatment [4]. In this study, we found that SUVmax-LN/Sp/Li in the M-HLH group was significantly higher than that in the NM-HLH group, and the malignant disease should be considered when SUVmax-LN >4.41 or SUVmax-Sp >2.785. Previous studies have found that the cut-off value of SUVmax-LN in distinguishing non-malignant disease and malignant disease was 3.3, while the cut-off value of SUVmax-Sp was 4.8 [4]. Compared with previous reports, the SUVmax-LN cut-off value was slightly higher, while the SUVmax-Sp cut-off value was slightly lower. At the same time, this study also found that SUVmax-Li and SUVmean-Sp can also be used as an index to distinguish the non-malignant disease from malignant disease. We also should notice that although a higher SUVmax is more suggestive of a malignant process, there is still a significant overlap of the SUVmax (or mean) between malignant and non-malignant conditions. So, studies with larger sample sizes are required to provide a more accurate cut-off value.

This study also found that focal hypermetabolic lesions of extranodal organs were more likely to occur in M-HLH (*P*=0.011). There was a significant difference in non-physiological focal uptake of the intestinal tract, skin, and/or/or nasal mucosa between the two groups (*P*=0.041, 0.045,  and 0.041). In a study of adult sHLH, Wang et al. found that hypermetabolism of lymph nodes and bone marrow in the malignant disease group was higher than that in the non-malignant disease group [4]. There is no significant difference between the two groups in this study, inconsistent with previous studies. The difference may result from the high physiological uptake in children's cervical lymph nodes and the large proportion of EBV infection-related lymphoproliferative diseases (EBV-LPD) in children's HLH. As LPD is often associated with multiple enlarged lymph nodes with increased FDG uptake, as well as focal hypermetabolism sometimes, it is challenging to differentiate LPD from lymphoma [11]. In this study, two patients with intestinal involvement were pathologically diagnosed with Hodgkin's lymphoma and non-Hodgkin's lymphoma. Moreover, in the 7 patients with skin and/or nasal mucosa involvement, 2 were diagnosed with non-Hodgkin's lymphoma, 1 was diagnosed with non-Hodgkin's lymphoma, and 1 was diagnosed with subcutaneous panniculitis T-cell lymphoma. It is suggested that when there is focal extranodal organ involvement, especially the non-physiological focal uptake of intestinal, skin, and/or nasal mucosa, the possibility of a malignant disease should be considered.

According to the current HLH-04 diagnostic criteria of the Histiocyte Society, the diagnosis of HLH mostly depends on laboratory examination. Previous studies have found that metabolic parameters of ^18^F-FDG PET/CT may be related to some laboratory parameters. Pak et al. [12] found that spleen FDG uptake is related to inflammatory reaction and immune response. Yang et al. studied the bone marrow FDG uptake in 34 cases of lymphoma-HLH and confirmed that bone marrow uptake could reflect the level of cytokine storm [13]. This study found that IFN- *γ* was positively correlated with SUVmax-BM/Li/Sp and SUVmean- BM/Li/Sp.

In contrast, serum ferritin, sCD25, IL-6, and IL-10 were positively correlated with SUVmax-Sp and SUVmean-Sp. HLH was mainly caused by excessive activation of macrophages and high expression of glucose transporters in activated macrophages and lymphocytes, resulting in an increased FDG uptake [14]. IFN- *γ* can lead to the increased FDG uptake in monocytes [15]. Cytokines such as IL-6 and IL10 can lead to the systemic immune response [16]. Like other studies, cytokine storms, especially inflammatory cytokines such as IFN-*γ*, IL-6, and IL-10, lead to the hypermetabolism of the spleen, liver, and bone marrow [17]. Inflammatory cytokines infiltrate the spleen and liver and lead to excessive activation of macrophages, and IL-6 and IL-10 can also lead to bone marrow proliferation. Kim et al. [18] found that FDG uptake in spleen and bone marrow is related to CRP and neutrophil count. Some studies have also found that high bone marrow uptake is only related to WBC [19]. The relationship between FDG uptake of bone marrow, liver, spleen, and blood cell level is still controversial. Its negative correlation may be related to bone marrow hyperplasia and extramedullary hematopoiesis activity caused by cytopenia in peripheral blood.

At the same time, this study also found that the role of ^18^F-FDG PET/CT in the diagnosis of CNS involvement is limited. This is consistent with previous reports that ^18^F-FDG PET/CT is less sensitive than MRI detecting CNS abnormalities. MRI can show early leptomeningeal and perivascular space lymphocyte infiltration, calcification, and necrosis of putamen, internal capsule, thalamus and dentate nucleus, white matter demyelination, brain atrophy, and ventricular dilatation [20]. Among the 14 patients with CNS involvement, only 2 cases showed hypermetabolic lesions on PET/CT. One patient was diagnosed with intracranial non-Hodgkin's lymphoma by craniotomy biopsy. Another patient diagnosed with EBV-HLH showed multiple hypermetabolic lesions in the occipital, temporal lobe, and cerebellum, which disappeared after hematopoietic stem cell transplantation. In the other 12 false-negative patients, 7 cases had abnormal signals on MRI, 4 cases were diagnosed by cerebrospinal fluid, and clinical manifestations diagnosed 1 case. Considering that the CNS involvement of HLH is different from another disease with CNS involvement, such as leukemia and lymphoma, nervous system symptoms may be secondary to the neurotoxic effects. Some chemokines or cytokines secreted by inflammatory cells may occur before cytotoxic T lymphocytes and macrophages infiltrate the brain, leading to parenchymal necrosis [21]. However, from another point of view, two ^18^F-FDG PET/CT positive patients were clinically confirmed to have CNS involvement, indicating that ^18^F-FDG PET/CT has a high specificity for the involvement of the CNS in children with sHLH.

Due to the complex etiology of sHLH, the value of ^18^F-FDG PET/CT in predicting HLH prognostic is unclear, and few articles refer to it. Some studies have pointed out that PET metabolic parameters or ratios such as SUV BM and SUV Sp, SUV BM/Li ratio, and SUV Sp/Li ratio can be used to predict the prognosis of patients with HLH [18]. As we all know, the prognosis of malignant disease-related HLH was much worse than that of non-malignant disease-related HLH, and different diseases need different treatment regimens after HLH has been controlled [22]. This study focuses on EBV-HLH, the most common sHLH in children, to exclude other confounding factors. It was found that almost all SUV parameters of bone marrow, including SUVmax-BM, SUVmax-BM, SUV BM/LiBG ratio, and SUV BM/M ratio, were significantly correlated with the prognosis of EBV-HLH in children. The higher level of SUV of bone marrow was associated with poorer treatment response in 2 weeks, more inferior OS, and EFS. The increased FDG uptake in bone marrow might be due to the activation of the immune system, the proliferation of T cells and macrophages, and the impaired regulation of immune response, but the exact mechanism was unknown. The small sample size might affect the statistical results, but it had a reference value for evaluating the prognosis of EBV-HLH in the future. Previous studies have found that spleen FDG uptake was very important for the prognosis of patients with sHLH. Regardless of the malignant degree of the primary disease, the SUV Sp/M ratio was an independent factor affecting the prognosis of HLH [23]. Hypermetabolism of the spleen might also predict inflammation, which was associated with poor prognosis and decreased survival in many types of cancer and HLH patients [24]. However, we did not find the relationship between spleen FDG uptake and prognosis.

This study had some limitations. First, this study was a retrospective study with small sample size. Second, this study had a selection bias because the children undergoing ^18^F-FDG PET/CT might be predisposed to the malignant tumor. In the study, there was significant heterogeneity of age, living environment, financial situation, and treatment preference, affecting patients' treatment effect and prognosis.

## 5. Conclusions

This study showed that ^18^F-FDG PET/CT metabolic parameters have a specific value in identifying the underlying etiology of sHLH in children. The presence of focal hypermetabolic lesions of extranodal organs indicates a high possibility of malignant diseases. Moreover, ^18^F-FDG PET/CT may be helpful to evaluate the prognosis of EBV-HLH. In the future, multicenter and large sample clinical studies need to be carried out to explore more accurate SUV indicators to further evaluate the prognosis of sHLH patients and establish an exact prognostic prediction model.

## Figures and Tables

**Table 1:** Clinical manifestations and laboratory results.

Items	Data	Reference
Clinical manifestations	Number (percent)	
Fever	66 (100.0%)	—
Hepatomegaly	43 (65.1%)	—
Splenomegaly	55 (84.8%)	—
Hemophagocytosis (bone marrow)	45 (68.2%)	—
Laboratory examination	Median (range)	
Absolute neutrophil count (10^9^/L)	0.98 (0.18–14.59)	1.2–7.0
Platelet (10^9^/L)	102.5 (1–319)	100.0–300.0
Hemoglobin (g/L)	93.5 (57–130)	110–160
Fibrinogen (g/L)	1.8 (0.55–4.91)	2.0–4.0
Serum ferritin (ng/mL)	1425.55 (13.20–139139.00)	6.00–159.00
Triglyceride (mmol/L)	2.54 (0.73–6.48)	0.40–1.70
sCD25 (pg/mL)	23407 (165–218875)	＜6400
NK cell activity (%)	15.74 (8.31–26.59)	＜15.10
EBV-DNA (whole blood) (×10^5^Copies/mL)	1.23 (0.005–245)	<5 × 10^2^
EBV-DNA (plasma) (×10^3^Copies/mL)	2.22 (0.50–7280.00)	<5 5810^2^
IFN-*γ* (pg/mL)	20.10 (0.00–2703.98)	1.60–17.30
TNF-*α* (pg/mL)	0.90 (0.00–51.59)	1.60–17.30
IL-6 (pg/mL)	24.78 (1.98–2500.00)	1.70–16.60
IL-10 (pg/mL)	29.49 (0.00–744.71)	2.60–4.90

sCD25: soluble cluster of differentiation 25; NK: natural killer; EBV: Epstein–Barr virus; IFN: interferon; TNF: tumor necrosis factor; and IL: interleukin.

**Table 2:** ^18^F-FDG PET/CT metabolic parameters.

PET parameter	Median (range)
SUVmax-M	0.98 (0.59–1.59)
SUVmean-M	0.81 (0.49–1.28)
SUVmax-LiBG	1.59 (0.93–5.06)
SUVmean-LiBG	1.34 (0.79–4.00)
SUVmax-Sp	2.01 (0.96–10.84)
SUVmean-Sp	1.67 (0.00–10.10)
SUVmax-Li	1.62 (0.94–12.60)
SUVmean-Li	1.34 (0.79–8.47)
SUVmax-LN	2.55 (0.44–20.40)
SUVmean-LN	2.13 (0.40–14.47)
SUVmax-BM	3.19 (1.77–16.99)
SUVmean-BM	2.90 (0.00–14.50)

SUV max: maximum standard uptake value; SUV mean: mean standard uptake value; M: mediastinum; LiBG: liver background; Sp: spleen; Li: liver; LN: lymph nodes; and BM: bone marrow.

**Table 3:** Comparison of focal hypermetabolism between M-HLH and NM-HLH group.

Organ with focal metabolic hypermetabolism	M-HLH (*n* = 13)		NM-HLH (*n* = 49)		*x* ^2^	*P* value
	Yes	No	Yes	No		
**Lymph nodes**	11	2	41	8	<0.001	<1.000
**Extranodal organs**	9	4	15	34	6.458	0.011^*∗*^
Spleen	1	12	6	43	<0.001	<1.000
Liver	2	11	3	46	—	0.280
Bone marrow	5	8	6	43	3.209	0.073
Intracranial	2	11	2	47	—	0.191
Lung	3	10	2	47	—	0.058
Intestinal	2	11	0	49	—	0.041^*∗*^
Renal	2	11	4	45	—	0.597
Adrenal	1	12	1	48	—	0.378
Skin and/or nasal mucosa	4	9	3	46	4.014	0.045^*∗*^
Central nervous system	1	12	1	48	—	0.378
Muscle	0	13	2	47	—	<1.000

M-HLH: malignancy-associated HLH and NM-HLH: non-malignancy-associated HLH.

## Data Availability

The data used to support the findings of the study are available from the corresponding author upon request.
